# The Law of Attrition

**DOI:** 10.2196/jmir.7.1.e11

**Published:** 2005-03-31

**Authors:** Gunther Eysenbach

**Affiliations:** ^1^Centre for Global eHealth InnovationUniversity Health NetworkToronto ONCanada

**Keywords:** Internet, clinical trials, longitudinal studies, patient dropouts, survival analysis

## Abstract

In an ongoing effort of this Journal to develop and further the theories, models, and best practices around eHealth research, this paper argues for the need for a “science of attrition”, that is, a need to develop models for discontinuation of eHealth applications and the related phenomenon of participants dropping out of eHealth trials. What I call “law of attrition” here is the observation that in any eHealth trial a substantial proportion of users drop out before completion or stop using the appplication. This feature of eHealth trials is a distinct characteristic compared to, for example, drug trials. The traditional clinical trial and evidence-based medicine paradigm stipulates that high dropout rates make trials less believable. Consequently eHealth researchers tend to gloss over high dropout rates, or not to publish their study results at all, as they see their studies as failures. However, for many eHealth trials, in particular those conducted on the Internet and in particular with self-help applications, high dropout rates may be a natural and typical feature. Usage metrics and determinants of attrition should be highlighted, measured, analyzed, and discussed. This also includes analyzing and reporting the characteristics of the subpopulation for which the application eventually “works”, ie, those who stay in the trial and use it. For the question of what works and what does not, such attrition measures are as important to report as pure efficacy measures from intention-to-treat (ITT) analyses. In cases of high dropout rates efficacy measures underestimate the impact of an application on a population which continues to use it. Methods of analyzing attrition curves can be drawn from survival analysis methods, eg, the Kaplan-Meier analysis and proportional hazards regression analysis (Cox model). Measures to be reported include the relative risk of dropping out or of stopping the use of an application, as well as a “usage half-life”, and models reporting demographic and other factors predicting usage discontinuation in a population. Differential dropout or usage rates between two interventions could be a standard metric for the “usability efficacy” of a system. A “run-in and withdrawal” trial design is suggested as a methodological innovation for Internet-based trials with a high number of initial dropouts/nonusers and a stable group of hardcore users.

## The Law of Attrition (Or: Why Do eHealth Users Discontinue Usage?)

**Figure 1:**
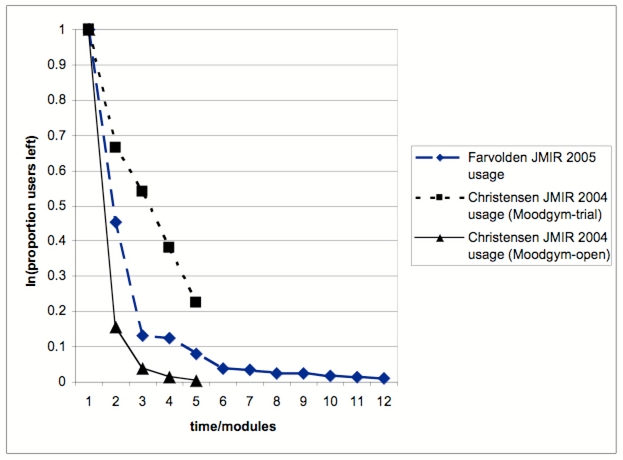
Nonusage attrition curves for two studies [1,2] published in this issue of the Journal of Medical Internet Research. Plotted are the number of completed modules from two Web-based interventions against the proportion of participants completing them. From the two Christensen/Moodgym curves, the upper one refers to a trial setting, while the other (lower one) refers to an “open” situation with casual Internet visitors.

 In this issue of the Journal, several excellent papers deal with the methodology of conducting Internet-based trials. Peter Farvolden and colleagues present an Internet-based evaluation of a panic disorder self-help Web program, struggling with a huge proportion of users discontinuing usage: only 12 out of 1161 (about 1%) completed the entire 12-week program [1]. A similar observation has been made previously by Christensen et al in her evaluation of Moodgym, a depression program with 5 modules, where only 97 out of 19607 (0.5%) participants completed all 5 modules in an “open “ setting, and 41 out of 182 (22.5%) completed all of them in a trial setting (Figure 1) [2,3]. Also in this issue, Wu et al report results from an exemplary study evaluating whether people would actually use (and continue to use) an innovative Internet-based communication and disease management platform requiring patients to enter different parameters and enabling them to exchange messages with clinicians online. He found that 26 out of 58 used it over a period of 3 months, only 16 patients continued to use the system after 12 months, 8 continued to use the system at 2 years, and 4 continued to used the system after 3 years [4]. Among the users, there also seemed to be a decline in the intensity of use, with a decrease in the number of messages entered by both patients and clinicians over time. These data are reminiscent of the experiences of Anhoj in a previous issue of the *Journal of Medical Internet Research*. Anhoj observed the contrast between users' positive perception of LinkMedica and their unwillingness to use the website for more than short periods. The primary reason for this was that LinkMedica “did not fit into their everyday lives.” [5] Finally, in this issue, is Jean-François Etter's landmark paper reporting results from one of the largest and perhaps best conducted Internet-based trials ever published to date [6]. He as well reports a considerable proportion of dropouts, with only 35% of the 11969 enrolled participants replying to the follow-up questionnaire. Amazingly, despite this huge loss-to-follow-up rate, the study still had enough statistical power to detect significant differences between the two interventions.

All these papers allude to a common problem: the law of attrition, as I call it, ie. the phenomenon of participants stopping usage and/or being lost to follow-up, as one of the fundamental characteristics and methodological challenges in the evaluation of eHealth applications.

While in most drug trials the intervention is “prescribed”, in studies involving information and communication technology usage of the intervention is mostly at the discretion of the participant and the participant has the option to discontinue usage very easily. In any longitudinal study where the intervention is neither mandatory nor critical to the participants' well-being, trial participants will be lost. Lack of compliance is usually not a major problem in drug trials, as participants are more closely supervised and sometimes experience observable and immediate health benefits in taking a drug. Thus, in drug trials, almost everyone in the intervention group will actually be getting the intervention (and receiving the same dose). In contrast, one of the fundamental methodological problems in eHealth trials is that in the intervention group a (sometimes substantial) proportion of people will not be using the intervention or using it sparingly [7]. It is difficult to measure an effect of an intervention if participants in the intervention group do not use the application.

In this paper I argue that a “science of attrition” is needed. Nonusage data *per se* should be of great interest to researchers, and attrition curves may be underreported and underanalyzed. Some theoretical models of attrition are proposed and I argue that by understanding and describing patterns and predictors for attrition and empirically verifying the proposed models, eHealth researchers may not only advance our understanding of the impact and uptake of eHealth interventions, but also contribute to the interdisciplinary field of diffusion research at large.

### Attrition Curves

When talking about attrition in longitudinal studies, we may actually refer to two different processes: the phenomenon of losing participants to follow-up (eg, participants do not return to fill in follow-up questionnaires), which I call *dropout attrition* here, and the phenomenon of nonusage, which I call *nonusage attrition*. Both may be closely related: often, high loss-to-follow-up rates indicate that a considerable proportion of participants have lost interest in the application and stopped using it. On the other hand, it may also be possible to have a low loss-to-follow-up rate, and still have participants not using (or infrequently using) the intervention (eg, in [2, 3]).

**Figure 2:**
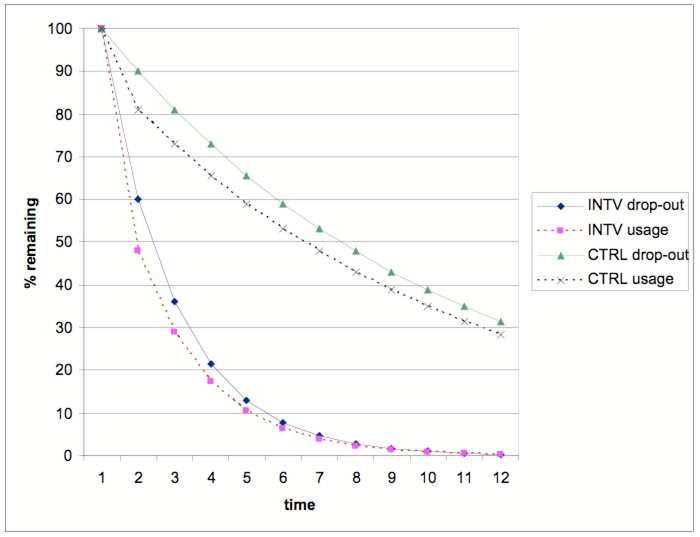
An example for logarithmic “attrition curves” in a hypothetical eHealth trial. In the intervention group (INTV), a proportion of participants will be lost to follow-up (INTV dropout), as will be in the control group (CTRL dropout). In addition, even within those not lost to follow up, there might be a proportion of nonusers

Thus, in any longitudinal eHealth study, we can draw two kinds of attrition curves: (1) proportion of users who are lost to follow-up over time, and (2) proportion of users who do not drop out (eg, who are still filling in questionnaires), but who are no longer using the application, plotted over time. My hypothesis is that the loss-to-follow-up attrition curve usually follows the nonusage attrition curve because a high proportion of loss to follow-up is a result of nonusage (“losing interest” is the underlying variable which explains both curves). In longitudinal studies with control groups, for example randomized trials, a third curve can be drawn to illustrate loss-to-follow-up rate in the comparison group. If the comparison group consists of providing another technological innovation, a fourth curve can be drawn to characterize nonusage of the control intervention (Figure 2).

**Figure 3:**
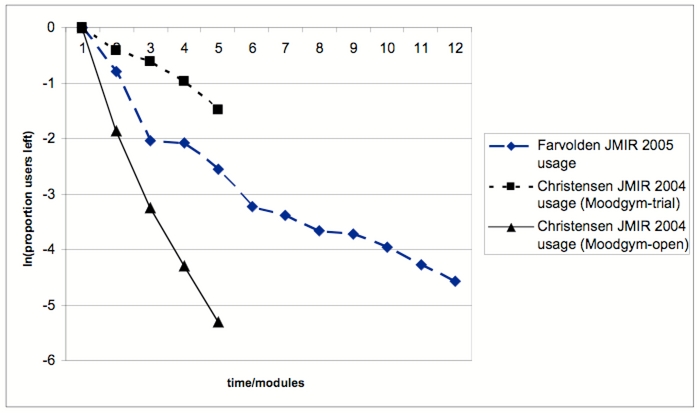
Attrition curves from Figure 1 on a logarithmic scale (y-axis is the natural logarithm of the proportion of users completing a module)

The hypothetical attrition curves in Figure 2 are logarithmic curves, and they are very similar to the actually observed attrition curves in the trials of Farvolden et al [1], Christensen et al [2], and Wu et al [4] (compare with Figure 1). In fact, when plotted on a logarithmic scale, the attrition curves from Figure 1 form almost straight lines (Figure 3).

### Nonusage Attrition: Diffusion of Innovation Reversed

The “science of attrition” can be seen as an application of (and contribution to) the theoretical framework of diffusion research. An eHealth intervention trial usually brings an innovation to participants. Everett M. Rogers defines innovation as “an idea perceived as new by the individual” and diffusion is “the process by which an innovation spreads.” [8] The model of diffusion of innovation proposed by Rogers was originally used by rural sociologists to study the diffusion of agricultural technologies in social systems. After its conception, an innovation spreads slowly at first — usually through the work of *change agents*, who actively promote it — then picks up speed as more and more people adopt it. Eventually it reaches a saturation level, where virtually everyone who is going to adopt the innovation has done so.

In trials of efficacy of eHealth interventions we are usually starting with an enrolled population of 100% “intent-to-use” participants, who have already gone through a recruitment, selection and informed consent process, ie, all of them have, in principle, already agreed to use and “adopted” the intervention. However, as shown above, in many trials a considerable number of users may discontinue the intervention or (worse) drop out of the trial altogether — a reversal of the adoption process.

In his 550-page book about how new ideas spread and are adopted, Rogers spends a mere 5 pages on *reversal* of decisions to adopt an innovation, illustrating how little research has been done in this area. Empirical evidence in eHealth (and perhaps in other areas in health care, for example, the self-help and self-support area in general, as noted by Farvolden [1]) suggests that abandoning an innovation is a significant phenomenon, perhaps deserving more attention and research. The fact that reversals of decisions are frequent is acknowledged by diffusion scholars. Rogers cites a study among Wisconsin farmers showing that the rate of discontinuance was just as important as the rate of adoption in determining the level of adoption at any particular time, for in any given year there were as many discontinuers as there were first-time adopters.

Rogers calls the innovation adoption stage where people may reverse their decision the *confirmation stage*. In this stage, according to Rogers, “The individual … seeks reinforcement for the innovation-decision already made, and may reverse this decision if exposed to conflicting messages about the innovation.” If a dissonance is created, ie, a state of internal disequilibrium or uncomfortable state of mind evolves, the innovation may be abandoned.

Rogers distinguishes *disenchantment* from *replacement* discontinuance. *Replacement discontinuance* is a “decision to reject an idea in order to adopt a better idea that supersedes it”, eg, MP3 and iPod players replacing walkmans, email replacing postal mail. In the context of Internet-based medical studies, the next website with (perhaps better) content competing for the attention of the participant is only a few mouseclicks away [8], making replacement discontinuance a not unlikely event. *Disenchantment discontinuance* leads to a rejection because the individuals are dissatisfied. In health care, disenchantment and replacement often go hand in hand, as it is often not possible to simply drop an intervention without using a replacement. In an Web-based communication tool intervention such as the one described by Wu et al [4], electronic messaging can, for example, be replaced by phone calls or office visits.

### Factors Influencing Attrition

In the classical model of Rogers, the rate of adoption is positively related to several characteristics of the innovation as they are perceived by the members of the system in which the innovation is diffusing. These are

1. relative advantage, the degree to which the innovation is perceived to be superior to the idea that it replaces;
2. compatibility, the degree to which an innovation is perceived as being consistent with the existing values, past experiences, and needs of potential adopters;
3. complexity, the degree to which an innovation is perceived as difficult to understand and use;
4. trialability, the degree to which an innovation may be experimented with on a limited basis; and
5. observability, the degree to which the results of an innovation are visible to others.

These characteristics of the innovation also play a role in the decision to stop using an eHealth innovation and/or to drop out of an eHealth trial. For example, the innovation will be rejected if it is not perceived as creating any benefit (relative advantage) or if it has usability problems (complexity). However, there are further factors involved which are not related to the innovation itself but more to the environment and the trial setting. These factors, for example, expectation management before the trial or “push factors” such as reminders by the study team, influence the shape and slope (steepness) of the attrition curve. In Figure 1 (and Figure 3) it is interesting to see how “push” factors involved in conducting a randomized trial of MoodGym (eg, research assistants contacting participants) lead to a flatter attrition curve, compared to a less “pushy” environment with casual users in an “open trial” of MoodGym (compare top and bottom curves).

A more formal analysis of such curves, eg, with methods of survival curve analysis, may elicit metrics for different attrition rates and identify factors affecting the shape and slope of these curves. Some of these proposed (hypothetical) factors have been compiled in Table 1.

There will also be additional participant factors, for example, demographics, which influence attrition rates. Users with less formal education, lower socioeconomic status, and less change agent contact are more likely to discontinue innovations [8]. Rogers also claims that later adopters (laggards) are more likely to discontinue innovations than earlier adopters ([8], generalization 5-11, p. 191). In the eHealth trial context, this perhaps means that if a participant hesitates to participate this may be an early indicator for a potential dropout. The predictive value of such factors for discontinuing a trial with a specific eHealth intervention could be identified by statistical models such as proportional hazards regression analysis (Cox model), comparing for example the dropout curve of the control group against the dropout curve of the intervention group.

**Table 1:** Proposed (hypothetical) factors influencing nonusage attrition and dropout attrition in eHealth trials

**Factor**	**Impact on Nonusage Attrition Rate**	**Impact on Dropout Attrition Rate**
Quantity and appropriateness of information given before the trial, expectation management	Inappropriate information leads to unrealistic expectations which in turn leads to disenchantment discontinuance	Indirectly through nonusage (usage discontinuance leads to drop out)
Ease of enrolment (eg, with a simple mouseclick as opposed to personal contact, physical examination etc), recruiting the “right” users, degree of pre-enrolment screening	If the “wrong” participants are enrolled, ie, those who are less likely to use it, and willing to invest time, and for whom the intervention does not “fit”	The easier it is to enroll, the more users will later drop out if they realize that filling in questionnaires, etc creates more work than they thought. Also indirect via nonusage.
Ease of drop out / stop using it	The easier it is to stop using the application, the higher the nonusage attrition rate will be (and indirectly through dropouts)	The easier it is to leave the trial, the higher the attrition rate will be (and indirectly through nonusage)
Usability and interface issues	Usability issues obviously affect usage	Indirectly through nonusage (usage discontinuance leads to drop out)
“Push” factors (reminders, research assistants chasing participants)	Participants may feel obliged to continue usage if reminded (cave external validity)	Participants may feel obliged to stay in trial
Personal contact (on enrolment, and continuous contact) via face-to-face or phone, as opposed to virtual contact	Mainly indirectly via dropout	The more “virtual” the contact with the research team is, the more likely participants will drop out
Positive feedback, buy-in and encouragement from change agents and (for consumer health informatics applications) from health professionals / care providers	Participants may discontinue usage without buy-in from change agents. In particular, patients may stop using eHealth applications if discouraged (or no actively encouraged) by health professionals	Indirectly through nonusage (usage discontinuance leads to drop out)
Tangible and intangible observable advantages in completing the trial or continuing to use it (external pressures such as financial disadvantages, clinical/medical/quality of life/pain)	Yes	Yes
Intervention has been fully paid for (out-of-pocket expense)	If individuals have paid for an innovation upfront they are less likely to abandon it (as opposed to interventions paid on a fee-per-usage basis)	Indirectly through nonusage (usage discontinuance leads to drop out)
Workload and time required	Yes	eg, to fill in the follow-up questionnaires may create such a burden that participants drop out
Competing interventions	For example similar interventions on the web or offline can lead to replacement discontinuance	Indirectly through nonusage (usage discontinuance leads to drop out)
External events (9/11 etc)	These may lead to distractions and discontinuance, especially if the intervention is not essential	Indirectly through nonusage (usage discontinuance leads to drop out)
Networking effects/peer pressure, peer-to-peer communication, and community building (open interactions between participants)	Communities may increase or slow the speed with which an innovation is abandoned.	Communities may increase or slow dropout attrition.
Experience of the user (or being able to obtain help)	As most eHealth applications require an initial learning curve and organizational change, users have to overcome initial hurdles to make an application work. Experience/external help can contribute to overcoming these initial hurdles and help to see the “light at the end of the tunnel”	Indirectly through nonusage (usage discontinuance leads to dropout)

### Measuring and Reporting Attrition

**Figure 4:**
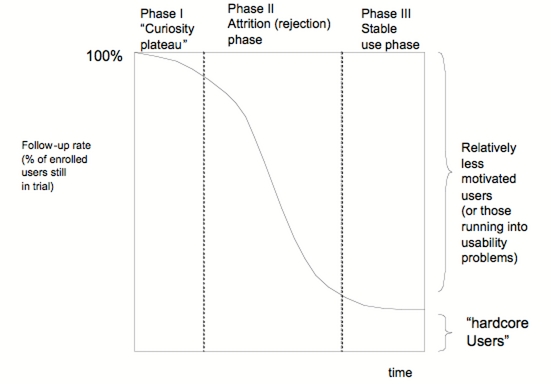
A (hypothetical) sigmoid attrition curve

When reporting the results of eHealth studies, a number of usage and dropout attrition metrics can (and should) be provided in addition to efficacy measures. Raw attrition proportions at different points in time should be reported and can be illustrated as attrition curves. The shape of the curve may indicate the underlying causes for attrition. A logarithmic curve, such as those in Figures 1 to 3, indicates a steady attrition with a constant proportion of users discontinuing use (or dropping out), similar to a probabilistic event. A sigmoid curve, such as the one illustrated in Figure 4, suggests a 3-phase process: an initial phase (Phase I) where participants out of curiosity initially stay in the trial (and use the eHealth application); Phase II where rejection and attrition set in, for example, because participants realize that the application does not meet their expectations; and Phase III where a stable user group (“hardcore users”) remains, who continue to use the application over extended periods of time. In contrast, an L-shaped curve (not shown, but similar to Phase II+III in Figure 4) reflects an initial rapid decline of participants and then a more steady group of “hardcore” users and/or trial participants who remain in the trial. This indicates an initial rapid weed-out process without preceding “curiosity plateau”, possibly because many of the enrolled participants were the wrong user group who lose interest quickly.

In addition to providing attrition curves, some summary metrics can be calculated. In biology, physics and economics the term “half-life” is used to measure “the time required for half of something to undergo a process” *(*Merriam-Webster Medical Dictionary). “Usage half-life” might be an useful measure to report for eHealth trials, indicating after how much time t50 (t10, t25…) will 50% (10%, 25% ….) of a volunteer user group have stopped using the application (As many applications hopefully have a slow attrition it might be more practical to report t10 or t25, where 10% or 25%, respectively, have been lost).

It is also interesting to formally compare different attrition curves, for example, a dropout attrition curve of intervention A against a dropout attrition curve of intervention B, evaluated in the same trial. For example, Christensen et al [3] report that after 6 weeks 89.3% remained in the control group, while only 74.7% in the Moodgym trial could be followed up, while the group using another intervention, Bluepages, had a follow-up rate of 84.9%, perhaps indicating more usability problems in the Moodgym application. If the attrition curve is logarithmic, it may be of advantage to report the logarithmic ratio ln(P_A_[tx]) / ln(P_B_[tx]) of two curves A and B (P[tx] being the proportions of users in group A or B still in trial and/or using the application after a certain time tx), because this ratio is constant across different points in time if the curve is logarithmic.

Further statistical comparisons across attrition curves can be done using Kaplan-Meier (survival curve) analysis and using Cox regression models.

### Dealing With Attrition: ITT and “Run-In and Withdrawal Design”

Dropout attrition is a threat to validity, because it may introduce a selection bias. For example, the intervention group may selectively lose more unmotivated people (who may have different outcomes due to the fact of being unmotivated) than the control group, and this differential dropout may lead to differences in outcomes measured among the remaining participants. An intention-to-treat (ITT) analysis, where all dropouts are assumed to have negative or neutral outcomes, is the only chance to avoid this bias. However, a high attrition rate and an intent-to-treat analysis greatly diminish the power to detect differences between groups (increasing the beta, ie, the chance that true differences are not measured).

**Figure 5:**
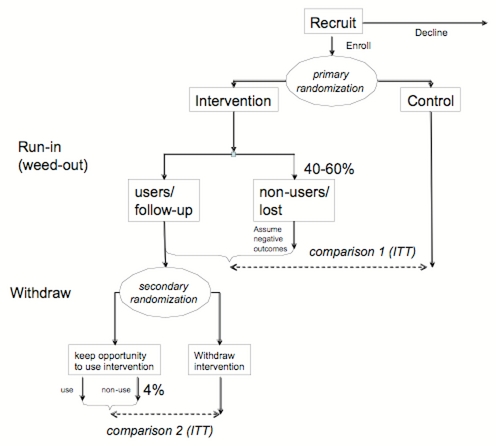
A proposed “run-in and withdrawal” design

ITT analysis could be combined with a method which I would call a “run-in and withdrawal design.” Here, the first phase of the trial (corresponding to Phase I and Phase II in Figure 4) is a “run-in and weed-out” period, where participants who will not want to use the application for an extended period of time are “weeded out” from the intervention group. This is followed (at the beginning of Phase III from Figure 4) by another randomization among the remaining actual users in the intervention group, which will be randomly split into those who can continue to use the application, and those from whom the application is withdrawn (Figure 5). The first evaluation after the run-in period will determine how many of the participants who originally intended to use the system actually used it, will determine the characteristics of the user group, and will give a conservative efficacy estimate based on a ITT comparison. For the second, the withdrawal phase, the intervention will be removed from half the users of the original intervention group. Comparing the withdrawal group with the nonwithdrawal group will then give a less conservative estimate for the effectiveness of the intervention – with the caveat of reduced generalizability, since this estimate is valid only for a subgroup of the population who actually end up using it.

Sadly, this design is only feasible if there is indeed a “hardcore” user group (ie, attrition virtually stops if the right users are found), if the outcomes are fully reversible, and if there are no learning or other carryover effects, such as in educational interventions. However, the proposed design is feasible for evaluating eHealth interventions which have a transient effect only for the duration in which they are used, such as evaluating email versus telephone communication with physicians, or evaluating access to electronic clinical guidelines, and so on.

### Conclusion: Overcoming Pro-Innovation Bias

The law of attrition may be a cause for publication bias, as authors with eHealth trials and high attrition rates may have difficulties in getting their work published. Journal editors and reviewers usually frown if they see substantial dropout rates. At the *Journal of Medical Internet Research*, studies with high dropout rates are welcome, because we know that in many cases discontinuance of eHealth innovations in a trial situation is a fact of life and worth reporting. Attrition data may give clues for real-life adoption problems.

The other reason that we see attrition rates so rarely analyzed in depth is that many investigators (in particular if they were involved in the development of an application) have an implicit pro-innovation bias, not expecting that an innovation will be rejected [8]. This leads to overlooking or underemphasizing discontinuance. As a consequence, Rogers notes that “We know too much about innovation successes and not enough about innovation failures.” For diffusion scholars, eHealth in particular presents a particularly rich field for studying rejected or discontinued innovations, and eHealth scholars might want to start directing their attention to attrition, uptake and diffusion measures with the same interest as they used to emphasize outcome efficacy.
